# Hopping species and borders: detection of *Bartonella* spp. in avian nest fleas and arctic foxes from Nunavut, Canada

**DOI:** 10.1186/s13071-020-04344-3

**Published:** 2020-09-14

**Authors:** Kayla J. Buhler, Ricardo G. Maggi, Julie Gailius, Terry D. Galloway, Neil B. Chilton, Ray T. Alisauskas, Gustaf Samelius, Émilie Bouchard, Emily J. Jenkins

**Affiliations:** 1 Department of Veterinary Microbiology, Western College of Veterinary Medicine, University of Saskatchewan, 52 Campus Drive, Saskatoon, SK S7N 5B4 Canada; 2 Intracellular Pathogens Research Laboratory, College of Veterinary Medicine, North Carolina State University, 1060 William Moore Drive, Raleigh, NC 27606 USA; 3 Department of Entomology, Faculty of Agricultural and Food Sciences, University of Manitoba, 12 Dafoe Road, Winnipeg, MB R3T 2N2 Canada; 4 Department of Biology, University of Saskatchewan, Science Place, Saskatoon, SK S7N 5E2 Canada; 5 Prairie and Northern Wildlife Research Centre, Wildlife Research Division, Environment and Climate Change Canada, 115 Perimeter Road, Saskatoon, SK S7N 0X4 Canada; 6 Snow Leopard Trust, 4649 Sunnyside Ave North, Suite 325, Seattle, WA 98103 USA

**Keywords:** Arctic fox, *Bartonella*, Disease ecology, Flea, Geese, Nunavut, Vector-borne disease, Wildlife, Zoonotic

## Abstract

**Background:**

In a warmer and more globally connected Arctic, vector-borne pathogens of zoonotic importance may be increasing in prevalence in native wildlife. Recently, *Bartonella henselae*, the causative agent of cat scratch fever, was detected in blood collected from arctic foxes (*Vulpes lagopus*) that were captured and released in the large goose colony at Karrak Lake, Nunavut, Canada. This bacterium is generally associated with cats and cat fleas, which are absent from Arctic ecosystems. Arctic foxes in this region feed extensively on migratory geese, their eggs, and their goslings. Thus, we hypothesized that a nest flea, *Ceratophyllus vagabundus vagabundus* (Boheman, 1865), may serve as a vector for transmission of *Bartonella* spp.

**Methods:**

We determined the prevalence of *Bartonella* spp. in (i) nest fleas collected from 5 arctic fox dens and (ii) 37 surrounding goose nests, (iii) fleas collected from 20 geese harvested during arrival at the nesting grounds and (iv) blood clots from 57 adult live-captured arctic foxes. A subsample of fleas were identified morphologically as *C. v. vagabundus.* Remaining fleas were pooled for each nest, den, or host. DNA was extracted from flea pools and blood clots and analyzed with conventional and real-time polymerase chain reactions targeting the 16S-23S rRNA intergenic transcribed spacer region.

**Results:**

*Bartonella henselae* was identified in 43% of pooled flea samples from nests and 40% of pooled flea samples from fox dens. *Bartonella vinsonii berkhoffii* was identified in 30% of pooled flea samples collected from 20 geese. Both *B. vinsonii berkhoffii* (*n* = 2) and *B. rochalimae* (*n* = 1) were identified in the blood of foxes.

**Conclusions:**

We confirm that *B. henselae*, *B. vinsonii berkhoffii* and *B. rochalimae* circulate in the Karrak Lake ecosystem and that nest fleas contain *B. vinsonii* and *B. henselae* DNA, suggesting that this flea may serve as a potential vector for transmission among Arctic wildlife.
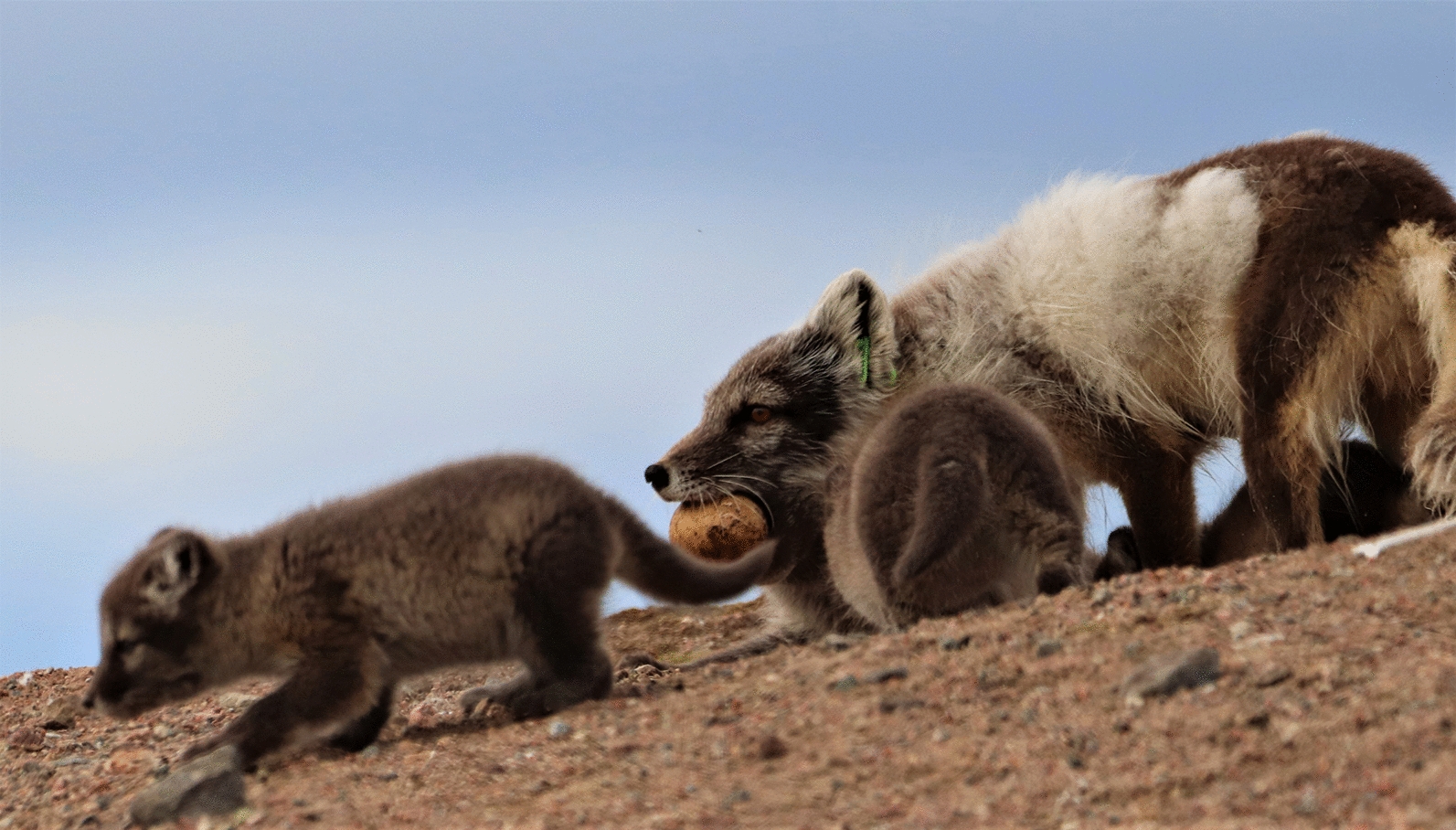

## Background

Migratory birds play a role in the global movement of pathogens. As the Arctic continues to warm, it is important to understand how movements of millions of migratory birds to Arctic nesting grounds and resulting avian host-parasite relationships may affect the transmission of vector-borne pathogens in Arctic ecosystems. *Bartonella* spp. are gram-negative fastidious intracellular bacteria that reside within erythrocytes, epithelial cells and macrophages [1–3]. Transmission can occur by (i) blood transfusions, (ii) animal scratches, and (iii) through a bite or (iv) the inoculation of feces from blood-feeding arthropods [4, 5]. A wide range of mammalian hosts develop chronic bacteremia, serving as sources of infection for arthropod vectors [6, 7]. The heterogeneity of host species has continued to grow with the discovery of *Bartonella* DNA in blood from loggerhead sea turtles (*Caretta caretta*), red-winged blackbirds (*Agelaius phoeniceus*) and northern mockingbirds (*Mimus polyglottos*), suggesting that non-mammalian species and their associated ectoparasites may play a role in the maintenance and dissemination of these pathogens [8, 9]. This group of bacteria has been found in numerous hematophagous arthropods, including ticks, sand flies, fleas, and lice [10, 11]. The list of potential vectors could also be larger than anticipated, as *Bartonella* DNA has recently been detected in avian-associated ectoparasites, including mites (*Dermanyssus prognephilus*), blow flies (*Protocalliphora sialia*) and fleas (*Ceratophyllus idius*) [12].

Previously, *Bartonella henselae* was isolated from the blood of 3 arctic foxes that were live-captured in the goose colony at Karrak Lake, Nunavut, Canada (67° 14ʹ N, 100° 15ʹ W) [13]. This was the first documentation of *B. henselae* in arctic foxes and was unexpected as suitable vectors have not been identified in Arctic ecosystems. Transmission cycles generally involve felids and their associated fleas, which are absent in the Arctic [14]. Arctic foxes in this region feed extensively on geese, eggs and goslings [15]. Blood-covered eggs in the colony were first noted in 1991 and were eventually attributed to a host-parasite relationship involving the nest flea, *Ceratophyllus vagabundus vagabundus* (Boheman, 1865) [16]. Since arctic foxes prey heavily on geese and their eggs, we hypothesized that nest fleas may function as a vector for transmission of *Bartonella* spp. at Karrak Lake. Therefore, we determined the prevalence of *Bartonella* spp. in fleas collected from geese, goose nests, and fox dens and blood clots from captured adult foxes. As *Bartonella* spp. are highly fastidious and difficult to culture, polymerase chain reaction analysis was used to detect DNA in fleas and foxes [17].

## Methods

This study was conducted from 2014 to 2018 at the large colony of Rossʼs (*Chen rossii*) and lesser snow geese (*Chen caerulescens caerulescens*) near Karrak Lake, Nunavut, Canada (67° 14ʹ N, 100° 15ʹ W). Karrak Lake is in the Queen Maud Gulf Migratory Bird Sanctuary and supports roughly 90% of the world’s population of Ross’s geese and 15% of the population of lesser snow geese during the summer months [18]. Population size of nesting Ross’s and snow geese at Karrak Lake increased from about 400,000 in 1993 to almost 1.2 million birds by 2010, reflecting broader continental population increases by both species of geese [19].

### Sample collection

**Fig. 1:**
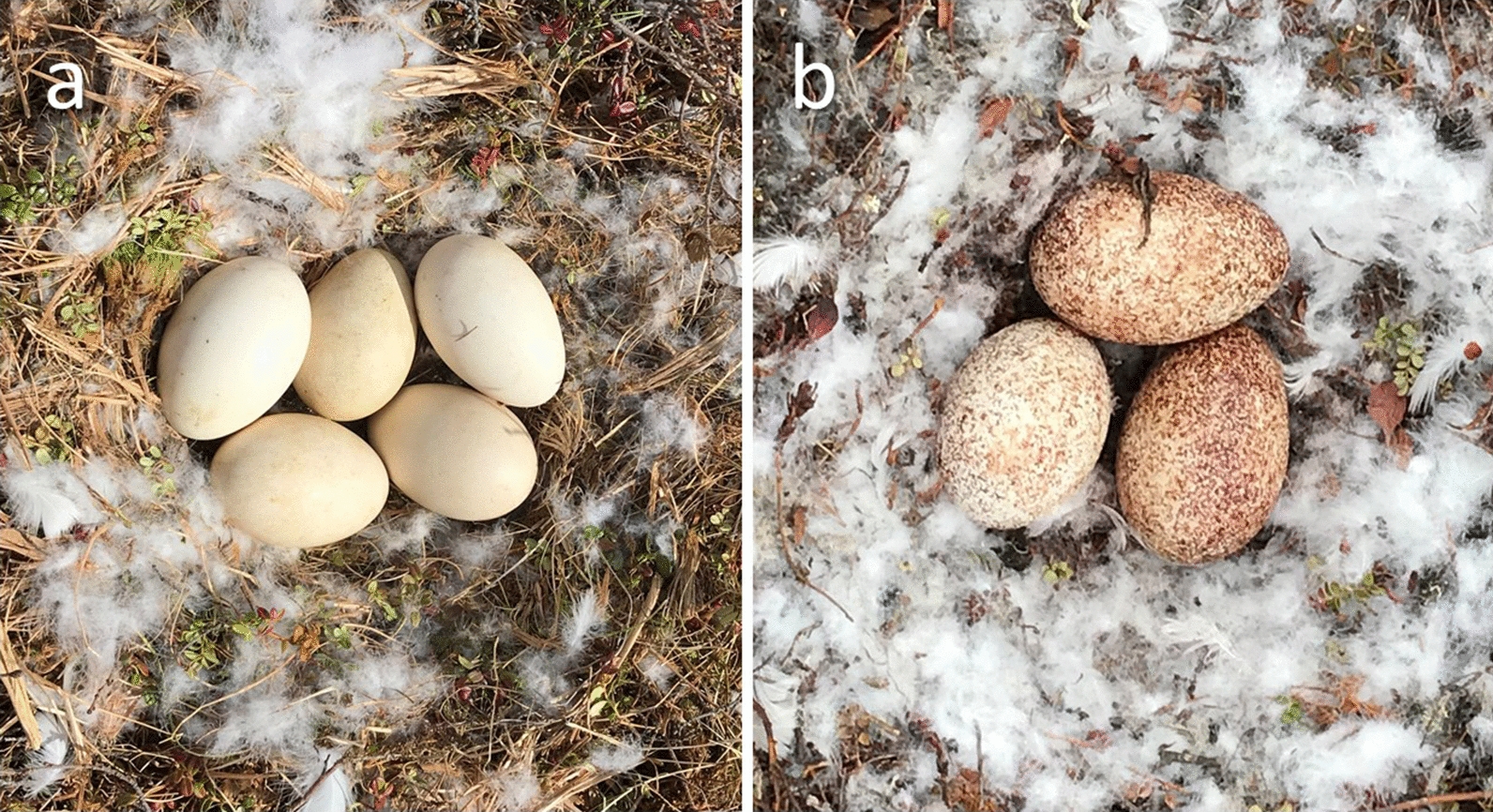
Photographs of eggs in an uninfested nest (**a**) and blood-covered eggs in a flea infested nest (**b**) in the Karrak Lake goose colony (Photos taken by Kayla Buhler in June 2019)

During the summer of 2014, fleas were collected from 5 fox dens located within the Karrak Lake colony by using a 25 × 25 cm square of white flannel placed on a plumber’s snake. This was extended into den entrances for a maximum of three meters and removed after 30 seconds. Similarly, 37 goose nests with evidence of flea infestation (Fig. 1) were sampled using a 25 × 25 cm square of white flannel placed over each incubating nest for one minute as per Harriman et al. [16]. These white flannel squares were placed individually into Ziploc bags and frozen overnight [20]. Fleas from each flannel square were then pooled and placed into a microcentrifuge tube containing 70% ethanol (one tube per den or nest). A total of 827 fleas were collected from goose nests and 86 fleas were collected from den sites.

Forty-eight Ross’s and 54 lesser snow geese were shot (see Ross et al. [21]) as they arrived at the nesting colony in early June 2018, placed into sealed clear plastic bags immediately, and held at ambient temperature overnight. Fleas found in the bag or on the goose were pooled for each host and placed into a microcentrifuge tube containing 70% ethanol. A total of 97 fleas were found on 19% of Ross’s geese (*n* = 9/48; 95% CI: 10–32%) and 20% of lesser snow geese (*n* = 11/54; 95% CI: 12–33%).

Fifty-seven adult arctic foxes were live-captured during May 2014–2018 as per Bouchard et al. [22]. Briefly, foxes were caught in box traps and sedated with 0.15–0.20 ml of Telazol® administered intramuscularly [23]. Ear tags were placed in both ears for future identification and blood was collected from the cephalic vein. Samples were then centrifuged, and sera and blood clots were stored separately in freezers until tested. Nest fleas are not active in May and have not been detected on adult foxes.

### Flea identification

As the process of mounting fleas on slides for species identification renders them unusable for molecular work, a subsample of roughly 25% (230 fleas from nests and 21 fleas from dens) were identified as *C. v. vagabundus* using morphological features as per Holland [24] and Lewis & Galloway [25]. All fleas collected from geese were morphologically identified to genus on gross inspection (*n* = 97), and one was mounted on a slide to verify that it was *C. v. vagabundus*. Given that there is no DNA sequence data available for this flea species in GenBank, DNA sequences were determined for part of the nuclear *28S* ribosomal RNA (rRNA) gene and the mitochondrial cytochrome c oxidase subunit 2 gene (*cox*2) for 2–3 representative specimens from dens and nests. Genomic DNA was extracted from the complete body of individual fleas using the DNeasy Blood & Tissue Kit (Qiagen, Hilden, Germany). PCRs were conducted using 25 µl reaction mixtures containing 10× Taq buffer with KCL (Fermentas, Vilnius, Lithuania), 25 mM MgCl_2_, 0.5 µl of dNTPs (Invitrogen, Carlsbad, USA), 0.25 µl of each primer, 0.1 µl of *Taq* DNA Polymerase (Fermentas) and 2 µl of genomic DNA. The primers used to amplify ~445-bp fragment of the *28S* rRNA gene were 28S-1 (5ʹ-ATA CGC CTT CGG CTT ATG CG-3ʹ) and 28S-2 (5ʹ-AAT AAG ACG CCC CGG GAT TG-3ʹ) [26], while the primers used to amplify ~615-bp fragment of the *cox*2 gene were COII-2a (5ʹ-ATA GAK CWT CYC CHT TAA TAG AAC A-3ʹ) and COII-9b (5’-GTA CTT GCT TTC AGT CAT CTW ATG-3ʹ) [27]. PCRs were conducted using the following conditions: 96 °C for 5 min followed by 30 cycles of 94 °C for 30 s, 55 °C for 30 s, 72 °C for 30 s, and a final extension step at 72 °C for 5 min. Amplicons were purified [28] and subjected to automated DNA sequencing. The DNA sequences of representative samples for each gene have been deposited in the GenBank database under the accession numbers MT470834 and MT471346.

### DNA extraction and PCR for *Bartonella*

DNA was extracted from pooled fleas corresponding to individual nests, dens, and geese and from fox blood clots using the DNeasy Blood & Tissue Kit from Qiagen (Table 1). Conventional (450–720 bp) as well as real-time qPCR (95–125 bp) targeting the 16S-23S rRNA intergenic transcribed spacer (ITS) region of *Bartonella* spp. was performed as previously described [6, 29]. For conventional PCR, screening of the *Bartonella* ITS region was performed using oligonucleotides 325s (5’-CTT CAG ATG ATG ATC CCA AGC CTT CTG GCG-3’) and 1100as (5ʹ-GAA CCG ACG ACC CCC TGC TTG CAA AGC A-3ʹ) as forward and reverse primers, respectively. Amplification was performed in a 25-µl final volume reaction containing 12.5 µl of Tak-Ex® Premix (Thermo Fisher Scientific, Waltham, USA), 0.25 µl of 30 µM of each forward and reverse primer (IDT® DNA Technology, Coralville, USA), 8 µl of molecular grade water, and 5 µl of DNA from each sample tested. PCR negative controls were prepared using 5 µl of DNA from blood of a healthy dog. Positive controls were prepared using 5 µl of genomic DNA from *B. henselae* SA2 (stock 0.001 pg/ µl). Conventional PCR was performed under the following conditions: 95 °C for 2 min followed by 55 cycles of 94 °C for 15 s, 66 °C for 15 s, 72 °C for 18 s, and a final extension step at 72 °C for 1 min. Products were analyzed by 2% agarose gel electrophoresis and then sequenced to establish species strain identification.

**Table 1:** Total number of fleas collected and pooled for PCR analysis

Sample	No. of sites/hosts	No. of fleas collected	No. of fleas analyzed (PCR)	No. of pooled samples	No. of fleas per pool
Nests	37	827	175	37	1–5
Dens	5	86	26	5	1–10
Geese	102	97	96	20^a^	1–5
Foxes	57	0	na	na	na

^a^20 of the 102 geese had fleas

*Abbreviation*: na, no fleas collected

For real-time qPCR, oligonucleotides 325s (5’- CTT CAG ATG ATG ATC CCA AGC CTT CTG GCG-3’) and 543as (5’-AAT TGG TGG GCC TGG GAG GAC TTG-3’) were used as forward and reverse primers and oligonucleotide BsppITS438probe (5’-FAM-GGT TTT CCG GTT TAT CCC GGA GGG C-BHQ1-3’) as Taqman probe. Amplification was performed in a 25-µl final volume reaction containing 12.5 µl of SsoAdvanced™ Universal Probes Supermix (Bio-Rad, Hercules, USA), 0.2 µl of 100 µM of each primer and probe (IDT® DNA Technology), 7.5 µl of molecular-grade water, and 5 µl of DNA from each sample tested. PCR negative controls were prepared using 5 µl of DNA from blood of a healthy dog. Positive controls were prepared by using 5 µl of DNA from a serial dilution (using dog blood DNA) of *B. henselae* genomic DNA equivalent to 0.1, 0.01 and 0.001 pg/ µl. Quantitative PCR was performed in CFX96® (Bio-Rad) under the following conditions: 95 °C for 3 min, followed by 45 cycles of 94 °C for 10 s, 68 °C for 10 s, 72 °C for 10 s, and a final extension step at 72 °C for 30 s. Positive amplification was assessed by analysis of detectable fluorescence *vs* cycle values and positive products were sent for sequencing to establish species strain identification.

## Results

There were no significant morphological differences between fleas collected from nests, dens, and geese. Amplicons of the expected size were obtained for all flea and blood samples subjected to PCR, whereas no products were obtained for the negative control samples. DNA sequences of part (436 bp) of the *28S* rRNA gene were obtained for three flea specimens originating from dens and nests. There was no intraspecific variation in DNA sequence. These sequences were also 100% identical to the *28S* sequences of *C. petrochelidoni* (GenBank: EU336152) and *C. gallinae* (GenBank: EU336148). The *cox*2 sequences (615 bp) obtained for two specimens of *C. v. vagabundus* (one from a den and one from a nest) were identical but differed at 18–41 bp (similarity of 94–97%) from the *cox*2 sequences of *C. hirundinis*, *C. gallinae*, *C. petrochelidoni* and *C. rusticus* (GenBank: KM8900834, KM8900832, AF424006 and KM8900836, respectively).

DNA from *Bartonella* spp. was amplified in 43% of pooled flea samples collected from individual nests (*n* = 16/37; 95% CI: 29–59%) and 40% of pooled flea samples from individual den sites (*n* = 2/5; 95% CI: 12–77%). Following sequencing, all *Bartonella* ITS amplicons from fleas collected in 2014 were identified as *B. henselae* (Table 2). Alignment analysis indicated that positive flea samples had 100% homology to *B. henselae* strain SA2 (GenBank: AF369529).

**Table 2:** Overall prevalence of *Bartonella* in fleas and foxes from Karrak Lake, Nunavut

Sample	*Bartonella* DNA detected	Prevalence (%)	95% CI (%)	% Homology (GenBank ID)
Fleas from nests	*B. henselae* SA2	43	29–59	100 (AF369529)
Fleas from dens	*B. henselae* SA2	40	12–77	100 (AF369529)
Fleas from geese	*B. vinsonii berkhoffii*	30	15–52	100 (DQ059763, DQ059765)
Arctic fox blood	*B. vinsonii berkhoffii*	4	0–12	98.8 (DQ059763, DQ059765)
Arctic fox blood	*B. rochalimae*	2	0–9	97.5 (KU292577)

DNA extracted from fleas collected from harvested geese (9 Ross’s geese and 11 lesser snow geese) and blood clots from foxes failed to amplify on conventional PCR using primers targeting the entire ITS region. A qPCR targeting 150–220 bp of the ITS region amplified *Bartonella* spp. DNA in 5% of fox samples (*n* = 3/57; 95% CI: 2–14%) and 30% of pooled flea samples from geese (*n* = 6/20; 95% CI: 15–52%). This includes fleas from two Ross’s geese (*n* = 2/9; 95% CI: 6–55%) and four lesser snow geese (*n* = 4/11; 95% CI: 15–65%). Quantification cycle (Cq) values were in the range of 34.66–38.51. ITS amplicons from fleas were identified as *B. vinsonii berkhoffii*, exhibiting 100% homology to genotype II or IV. Unfortunately, the small size of the amplicon available for sequencing was not enough to differentiate among the two genotypes (DQ059763 and DQ059765). Similarly, blood samples from three foxes were positive for *Bartonella via* qPCR. The two positive samples collected in 2014 and 2016 were 98.8% homologous with the same *Bartonella* species detected in fleas (*B. vinsonii berkhoffii* DQ059763 and DQ059765). Meanwhile, a third fox blood sample from 2018 was positive, with 97.5% homology to *B. rochalimae* (GenBank: KU292577).

## Discussion

This study verifies that *Bartonella* spp. circulate in the remote terrestrial ecosystem at Karrak Lake and suggests that nest fleas may serve as a potential vector for transmission between birds and mammals. As with previous studies, fleas collected from nests and geese were identified as *C. v. vagabundus* [16, 30]. Fleas collected from fox dens were also identified as *C. v. vagabundus*. Though all fleas were not individually identified in this study, the morphology, host, location, prior documentation at Karrak Lake, and relative scarcity of ectoparasites in the Arctic suggests that all specimens were most likely *C. v. vagabundus*. *cox*2 sequences for two fleas (one from a den and one from a nest) were identical, indicating that fleas may transfer from geese or nests to foxes during foraging efforts [15]. *Ceratophyllus* spp. have sporadically been reported on domestic dogs [31, 32], but this is the first report of a *Ceratophyllus* species in the dens of arctic foxes.

Fleas were found on geese that were shot on the southern border of the colony during arrival in spring, suggesting that this host-parasite relationship facilitates the transport and transmission of nest fleas and associated pathogens through Arctic terrestrial ecosystems. The known range for *C. v. vagabundus* is limited to the Arctic, and fleas found on geese may have originated from southern parts of Nunavut [24]. Over the last few decades, there has been a contemporaneous exponential growth in continental population size of both snow and Ross’s geese that nest at Karrak Lake [19]. This surge in abundance of migrating geese affects nest density in the colony, which may increase transmission of nest parasites between geese by decreasing distance between hosts [16]. These characteristics highlight the role of migratory geese in the dissemination of flea-borne pathogens during migration and nesting.

**Fig. 2:**
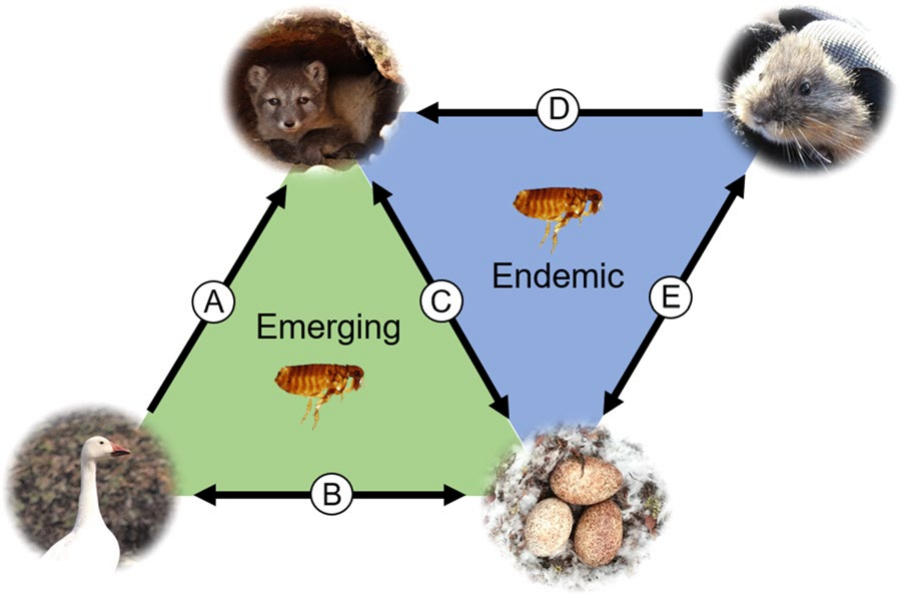
Hypothesized mechanisms of *Bartonella* transmission in a terrestrial Arctic ecosystem. **a** Goose carcasses that are brought to dens may expose fox adults and kits to infected fleas. **b** Migrating geese may introduce *Bartonella* species from southern latitudes to fleas overwintering in nest material in the Arctic. Alternatively, nest fleas may expose geese to *Bartonella* if the bacteria can be maintained over the harsh arctic winter. **c** Collecting and caching eggs may result in transmission of *Bartonella* to foxes *via* nest flea bites or exposure to flea dirt on the surface of eggs. Alternatively, fleas may collect infected blood meals from foxes. **d** Predation of Arctic rodents may expose foxes to rodent fleas carrying *Bartonella* species. **e** Rodents may visit newly abandoned nests to consume egg remnants and may expose nest fleas to *Bartonella* species. Rodents may also be exposed to *Bartonella* species *via* flea feces or bites in nests

Fleas collected from dens and goose nests during the study contained *B. henselae* DNA, which suggests that previous reports by Mascarelli et al. [13] of *B. henselae* in arctic foxes from Karrak Lake may have resulted from a transient infestation with nest fleas or exposure to flea feces during nest predation (Fig. 2). However, it is important to note that detection of *Bartonella* DNA in fleas does not provide definitive proof of vector competence, as arthropods may have ingested blood meals from infected hosts. Thus, directionality of transmission could not be determined (fleas and/or flea dirt may infect foxes, foxes may infect fleas, or both). Subsequent studies are needed to address this question. For example, blood-meal composition and the presence or absence of *Bartonella* DNA in flea feces on the surface of eggs would provide vital information about feeding habits and the potential for transmission during predation.

DNA of *B. vinsonii berkhoffii* was detected in fleas from 30% of infested geese. This species of *Bartonella* has been implicated in cases of clinical bartonellosis in dogs and evidence of infection has been found in a range of wild canids [33, 34]. Cq values were high (ranging from 34.66 to 38.51), indicating that the concentration of bacterial DNA was relatively low and that bacteremic blood may have been ingested long before fleas were collected. Prevalence of *Bartonella* spp. in fleas from nests and geese (43% for *B. henselae* and 30% for *B. vinsonii berkhoffii*) was high in relation to foxes (4% for *B. vinsonii berkhoffii* and 2% for *B. rochalimae* in the present study; 11% for *B. henselae* in Mascarelli et al. [13]), suggesting that fleas are likely to (i) be a competent vector or (ii) have frequent access to blood meals from a competent host. Experimental studies to document vector competence are required to distinguish between these two possibilities. Future work could determine whether DNA of *Bartonella* spp. is present in the blood and spleens of geese at Karrak Lake, as they provide the majority of the blood meals for the fleas.

The overall prevalence of *Bartonella* spp. DNA in arctic foxes was 5%, with all foxes exhibiting low levels of bacteremia (Cq values from 36.88 to 38.28). This suggests that the animals could have been sampled at a later stage of infection, consistent with exposure during the prior summer when vectors were active. Sequence analyses suggested that two species of *Bartonella* are present in this population: *B. vinsonii berkhoffii* (in two foxes) and *B. rochalimae* (in a single fox). These results, along with the study by Mascarelli et al. [13], verify that the same species of *Bartonella* are present in foxes and nest fleas (*B. henselae* and *B. vinsonii berkhoffii*) and supports the hypothesized mechanism of transmission between foxes and fleas during predation. The prevalence of *Bartonella* spp. in foxes suggests that they are unlikely to be maintenance hosts and that nest fleas may acquire blood meals from a more competent host species. However, it is important to note that adult foxes in this study were sampled in May before temperatures increased and that the prevalence in foxes may differ during months with peak insect activity. The detection of *B. rochalimae* in foxes may suggest that Arctic rodents and their associated fleas also play a role in the transmission of *Bartonella* above the treeline, as *B. rochalimae* DNA has been documented in rodents and gray foxes (Fig. 2) [35, 36]. Fleas have not been detected on adult arctic foxes during the month of May, and further studies could determine ectoparasite abundance and prevalence of *Bartonella* spp. in juvenile foxes captured during the summer months when birds are nesting, and insects are active.

Fleas that exhibit generalist tendencies may harbour multiple species of *Bartonella*, and the detection of *B. henselae* and *B. vinsonii berkhoffii* in nest fleas suggests that co-infections may be possible [37]. Based on previous publications, coinfections may limit the detection of different species of *Bartonella* [6]. For example, when *B. henselae* is present in equal numbers to *B. vinsonii*, only *B. henselae* will be amplified to quantities that can be sequenced. Therefore, both species of *Bartonella* may have been present in 2014 and 2018. However, the concentration of *B. henselae* in 2014 may have masked the presence of *B. vinsonii*. Alternatively, this observation could result from variation in blood meals from infected hosts between years.

Manifestations of disease following *Bartonella* spp. infection in arctic foxes, and wildlife in general, are poorly understood. Infections with *B. henselae*, *B. vinsonii berkhoffii* and *B. rochalimae* in domestic canids can range from subclinical bacteremia to severe illness, including lymphadenomegaly and endocarditis [35, 38]. These pathogens can result in the development of vasoproliferative lesions in dogs [39], and foxes may present with similar clinical symptoms and pathogenesis. Species of *Bartonella* that were identified during this study have zoonotic potential and may be transmitted to people that are in close contact with fleas and foxes, such as hunters, trappers, and biologists [40, 41]. *Bartonella* spp. and other zoonoses may be especially relevant in the Arctic where hunting and trapping are integral to both the culture and economy [42, 43].

## Conclusions

This study demonstrates that *B. henselae*, *B. vinsonii berkhoffii* and *B. rochalimae* are present in the Karrak Lake ecosystem. Our study suggests that migratory geese may play a role in the dissemination of flea-borne pathogens and that contact between foxes and nesting geese is likely to be involved in the transmission dynamics of *Bartonella* spp. in the Arctic. However, other sources of infection and the competence of *C. v. vagabundus* are unknown. Future studies may investigate flea blood-meal composition and the presence of *Bartonella* DNA in geese, flea feces on the surface of eggs, juvenile arctic foxes, and rodents. Addressing these questions will increase our understanding of the epidemiology of *Bartonella* spp. above the tree line, the role of migratory wildlife in the movement of pathogens, and the vulnerability of northern and remote regions to the emergence of vector-borne pathogens.

## Data Availability

The datasets supporting the conclusions of this article are available in the Zenodo repository http://doi.org/10.5281/zenodo.3905327. Representative newly generated sequences were deposited in the GenBank database under the accession numbers MT470834 and MT471346.
